# Multimodal Imaging of Orthotopic Mouse Model of Endometrial Carcinoma

**DOI:** 10.1371/journal.pone.0135220

**Published:** 2015-08-07

**Authors:** Ingfrid S. Haldorsen, Mihaela Popa, Tina Fonnes, Njål Brekke, Reidun Kopperud, Nicole C. Visser, Cecilie B. Rygh, Tina Pavlin, Helga B. Salvesen, Emmet McCormack, Camilla Krakstad

**Affiliations:** 1 Department of Radiology, Haukeland University Hospital, Bergen, Norway; 2 Section for Radiology, Department of Clinical Medicine, University of Bergen, Bergen, Norway; 3 Department of Clinical Science, University of Bergen, Bergen, Norway; 4 Centre for Cancer Biomarkers, Department of Clinical Science, University of Bergen, Bergen, Norway; 5 PET-centre, Department of Radiology, Haukeland University Hospital, Bergen, Norway; 6 Department of Pathology, Radboud University Medical Center, Nijmegen, The Netherlands; 7 Molecular Imaging Center, Department of Biomedicine, University of Bergen, Bergen, Norway; 8 Department of Obstetrics and Gynaecology, Haukeland University Hospital, Bergen, Norway; Northwestern University Feinberg School of Medicine, UNITED STATES

## Abstract

**Background:**

Orthotopic endometrial cancer models provide a unique tool for studies of tumour growth and metastatic spread. Novel preclinical imaging methods also have the potential to quantify functional tumour characteristics *in vivo*, with potential relevance for monitoring response to therapy.

**Methods:**

After orthotopic injection with luc-expressing endometrial cancer cells, eleven mice developed disease detected by weekly bioluminescence imaging (BLI). In parallel the same mice underwent positron emission tomography–computed tomography (PET-CT) and magnetic resonance imaging (MRI) employing ^18^F-fluorodeoxyglocose (^18^F-FDG) or ^18^F- fluorothymidine (^18^F-FLT) and contrast reagent, respectively. The mice were sacrificed when moribund, and post-mortem examination included macroscopic and microscopic examination for validation of growth of primary uterine tumours and metastases. PET-CT was also performed on a patient derived model (PDX) generated from a patient with grade 3 endometrioid endometrial cancer.

**Results:**

Increased BLI signal during tumour growth was accompanied by increasing metabolic tumour volume (MTV) and increasing MTV x mean standard uptake value of the tumour (SUV_mean_) in ^18^F-FDG and ^18^F-FLT PET-CT, and MRI conspicuously depicted the uterine tumour. At necropsy 82% (9/11) of the mice developed metastases detected by the applied imaging methods. ^18^F-FDG PET proved to be a good imaging method for detection of patient derived tumour tissue.

**Conclusions:**

We demonstrate that all imaging modalities enable monitoring of tumour growth and metastatic spread in an orthotopic mouse model of endometrial carcinoma. Both PET tracers, ^18^F-FDG and ^18^F-FLT, appear to be equally feasible for detecting tumour development and represent, together with MRI, promising imaging tools for monitoring of patient-derived xenograft (PDX) cancer models.

## Introduction

Endometrial cancer is the most common pelvic gynaecologic malignancy in industrialized countries, and the incidence is increasing [1]. Although about 75% of the patients are treated with tumour confined to the uterine corpus, 15–20% recur [2]. In patients with distant metastases or locally recurrent disease, the effect of the conventional systemic therapy is poor with reported median survival ranging from 7–12 months [3]. Thus, there is an urgent need to develop more efficient therapies for metastatic endometrial cancer.

Preclinical testing of drug efficacy has been reliant upon subcutaneously implanted tumours originating from human cancer cell lines or tumour biopsies into immunodeficient rodents [4]. Also for endometrial cancer, subcutaneous xenograft models have long been employed to explore effect of new treatments [5]. This model enables monitoring of tumour growth by visual inspection and palpation to monitor tumour growth. However, the subcutaneous model has important limitations including non-metastatic behaviour, thus lacking immediate relevance for humans [6]. Orthotopic xenograft models, whereby molecularly defined cancer cell lines or primary patient cells are surgically implanted into the organ of origin, induce disease that more accurately reflect human metastatic patterns and response to therapeutics. Orthotopic endometrial cancer models have been successfully developed [6–8]. This has provided a valuable research platform for studies of molecular and cellular mechanisms underlying tumour growth and metastatic spread in endometrial cancer [8–13].

A challenge in such models is still to accurately determine tumour growth and drug efficacy longitudinally. Bioluminescence imaging (BLI) represents one such useful preclinical imaging method for *in vivo* monitoring of tumour growth and metastases in endometrial cancer xenograft models from human cell lines, but requires that these are transfected with luciferase gene [8, 9, 13]. Patient derived tumour xenograft (PDX) models, which better mimic the corresponding human lesion and tumour growth (i.e. molecular type, stromal tissue interaction and three dimensional growth in relevant organ) represent a more reliable tool to predict response to chemotherapy [4]. Although methods are available to genetically manipulate PDX models *ex vivo*, such manipulation cause irreversible genetic changes distancing the models from the parental tumours [14]. BLI is therefore not an optimal method for PDX models [15]. Thus, additional *in vivo* preclinical imaging methods to identify and quantify orthotopic endometrial cancer xenograft progression and response to therapy, needs to be better explored to fully exploit orthotopic PDX endometrial cancer models.

Preclinical positron emission tomography-computed tomography (PET-CT) and magnetic resonance imaging (MRI) provide both anatomical and functional information from tumour tissue [15, 16]. These novel imaging methods have been shown to predict response to therapy in various xenografts models [16] such as in colorectal cancer [17] (based on ^18^F-FLT and ^18^F-FDG PET), breast cancer [18] (dynamic contrast-enhanced (DCE)-MRI and diffusion weighted imaging (DWI)) and in Ewing sarcoma [19] (whole body MRI and DWI). Characteristics for PET-CT or MRI findings in endometrial cancer orthotopic mouse models have not yet been reported, hence the feasibility of these novel imaging methods in monitoring tumour progression and metastatic spread in this setting is largely unknown.

This study presents characteristic preclinical imaging findings for *in vivo* BLI, PET-CT (with ^18^F-FDG and ^18^F-FLT) and MRI during tumour progression and metastatic spread in an orthotopic endometrial cancer model. These observed *in vivo* imaging findings are also related to the *ex vivo* BLI findings of single organs at necropsy and to histological characteristics for the corresponding tumour tissue.

## Material and Methods

### Ethics statement

For patient samples and information, all parts of the study have been approved according to Norwegian legislation, including the Norwegian Data Inspectorate, Norwegian Social Sciences Data Services, and the Western Regional Committee for Medical and Health Research Ethics, (NSD15501; REK 052.01). Participants gave written informed consent. All animal studies were approved by the Norwegian State Commission for Laboratory Animals (ID 4036) and performed according to the European Convention for the Protection of Vertebrates Used for Scientific Purposes.

### Cell lines and Retroviral transfection

The human endometrial cancer cell line Ishikawa was obtained from Sigma-Aldrich (St. Louise, MO, USA) and the cell authenticity was confirmed by Short Tandem Repeat (STR) profiling (IdentiCell, Denmark). Cells were kept in Minimal Essential Medium (MEM; Lonza, Basel, Switzerland) supplemented with 5% heat-inactivated Fetal Calf Serum (FCS; Sigma-Aldrich, St. Louis, MO, USA), 2 mM L-glutamine (Lonza, Basel, Switzerland), 1% non-essential amino acids (Lonza, Basel, Switzerland), penicillin 100 IU/ml and 100 μg/ml streptomycin (Lonza, Basel, Switzerland) at 37**°**C in a humidified atmosphere with 5% CO_2_. Ishikawa cells were stably transfected using retroviral infection as described previously [20, 21] using the luciferase expressing construct L192, combined with the tetracycline-regulated transactivator (tTA). Stably transfected Ishikawa^Luc^ cells were selected with 1 μg/ml puromycine (Sigma-Aldrich, St. Louis, MO, USA) and luciferase expression was confirmed by adding 2.5 mg/ml D-luciferin (Promega, Madison, WI, USA) before *ex vivo* optical imaging.

### Orthotopic endometrial cancer model

NOD-*scid* IL2Rgamma^null^ (NSG) mice were originally a gift from Prof. Leonard D Schultz at The Jackson Laboratory (Maine, USA) and bred at the Vivarium, University of Bergen, Norway. Female 6–8 weeks old were maintained under pathogen-free conditions with food and water provided *ad libidum*. Animals were kept on a 12 hours dark/night schedule at a constant temperature of 21°C and at 50% relative humidity. Prior to surgery animals received 0.1 mg/kg Buprenorphine hydrochloride (Temgesic, Reckitt Benckiser, Berkshire, UK) intramuscular, for analgesia. Mice were anaesthetised with 250 mg/kg tribromoethanol (Sigma-Aldrich, St. Louis, MO, USA) diluted in 2 methyl-2 butanol (Sigma-Aldrich, St. Louis, MO, USA) and placed on a heating pad in dorsal decubitus. Fur on the abdomen was clipped and skin disinfected with surgical iodine and 70% ethanol. A 1cm middle line incision was made in the lower abdomen (skin and muscles). The left uterine horn was exteriorized and 1x10^6^ of Ishikawa^Luc^ cells resuspended in 50 μl of Matrigel (BD Matrigel Basement Membrane Matrix, BD Biosciences, San Jose, CA) were injected directly into the endometrial cavity through the myometrium. A 0.3mm insulin syringe (Omnican 50, B-Braun, Melsungen, Germany) was used for the injection. The uterine horn was put back in the original position before muscles and skin was closed with 5–0 absorbable sutures. After the surgery the animals were placed in a warm environment and supervised until full recovery.

### Generation and maintenance of patient derived xenograft (PDX) model

A biopsy from the primary tumour of a 69 year old woman diagnosed with grade 3 endometrioid endometrial cancer was placed on ice until processing. Tissue was mechanically dissociated using sterile scalpels and sequentially filtered through a 40 μm pore filter (Fisher) and centrifuged at 900rpm for 4 min. The cell pellet was resuspended in Matrigel and orthotopic implantation in four mice was performed as described above. The mice (F1 generation) were monitored closely for visible signs of disease development and examined using PET CT when clinical signs of disease were presented. Mice were thereafter sacrificed, and a cell suspension from the primary tumour was prepared and implanted in the next generation of mice (F2). Samples for histological examination of tumour grade and type were taken in parallel. The new generation was monitored in a similar manner until presenting clinical signs of disease, when PET CT was performed. The mouse model is continuously rederived following the same protocol.

### Experimental set-up for multimodal imaging

#### Optical imaging

In total 15 mice were orthotopically injected with Ishikawa^Luc^ cells. All mice were subjected to weekly examination by bioluminescence to follow tumour growth and metastatic dissemination. Mice were injected intraperitoneally (i.p.) with D-luciferin (150 mg/kg) and anaesthetised with 3% isoflurane (Isoba Vet, Schering-Plough, Brussel, Belgium) 10 minutes before optical imaging using an In-Vivo FX PRO molecular imaging system (Carestream Health, Inc., Rochester, NY, USA). Total bioluminescence values were measured using manual Region of interest (ROI) of the whole abdomen using the Carestream MI software (Standard Edition, v.5.0.6.20, Carestream Health, Inc.). Three mice showed no BLI signal after four weeks, suggesting no tumour development in the uterus, possibly due to vaginal leakage of cells after surgery. One mouse died during anaesthesia for PET scan. These four mice were excluded from the experiment. The 11 remaining mice were monitored weekly and euthanized when moribund as defined by weight loss 10–15%, lethargy or ruffled fur. Ten minutes before necropsy, all mice were injected i.p. with D-Luciferin and organs were imaged *ex vivo* for evaluation of disease dissemination using Optix MX3 Small Animal Molecular Imager (ART Inc., Saint-Laurent, QC, Canada) supplied with Optix Optiview software. After BLI imaging, the tissue biopsies were fixed in 4% buffered formalin and embedded in paraffin before they were processed for histological analysis.

#### PET-CT

PET-CT was performed in all mice weekly from week 5/6–week 7/8 after injection of Ishikawa^Luc^ cells. In one of the mice PET-CT was also performed 12 and 13 weeks after Ishikawa^Luc^ cells injection. The PET-CT scans consisted of ^18^F-FLT PET only (n = 2), ^18^F-FDG-PET only (n = 3) or both ^18^F-FLT PET and ^18^F-FDG PET (n = 6). The last PET-CT examinations were performed 7 days (n = 1), 11 days (n = 3), 18 days (n = 1), 35 days (n = 2) and 49 days (n = 4) respectively before the mice were sacrificed, respectively. This approach was chosen in order to reduce the cost of the experiments as well as to minimize stress for individual animals also undergoing weekly BLI. For mice with slow tumour progression limited capacity of the PET-scanner precluded PET scanning immediately prior to sacrifice. For the PDX model, the PET-CT scans consisted of ^18^F-FLT PET in the F1 generation and ^18^F-FDG-PET in the F2 generation. PET-CT scans were performed using the integrated PET-CT scanner nanoScan PC PET/CT (Mediso Medical Imaging Systems Ltd, Budapest, Hungary) featuring spatial resolutions of 800 μm and 30 μm of the respective PET- and CT detector systems. The PET field of view (FOV) was 9.5 x 8 cm in axial and transaxial directions allowing whole-body imaging of mice. The PET detectors consist of LYSO crystals, and acquisition was performed in 1:5 coincidence and normal count mode. Mice were scanned simultaneously without prior fasting using a dual mouse bed with integrated system for anaesthesia and heating. Animals were anaesthetised using 3% sevoflurane (Sevoflo, Abbott, Illinois, USA) and ^18^F-FDG (mean dose of 7.3 ^+^/_-_1.6 MBq) was injected via the tail vein 30 seconds after the start of the PET scanning. Total scan time was 60 minutes, and the last 30 minutes was reconstructed into a static image. For ^18^F-FLT PET-CT mice were anesthetised and ^18^F-FLT (mean dose of 7.4^+^/_-_2.3 MBq) was injected via the tail vein 30 minutes prior to scanning. PET acquisition time was 30 minutes. For both tracers, a whole-body CT scan (helical projections with tube energy of 50 kvP, exposure time 300 ms, 720 projections, max FOV, binning 1:4) was performed for anatomical information and attenuation correction of PET images.

#### Reconstruction and post-processing of PET-CT data

The PET images were reconstructed using the supplier’s reconstruction algorithm Tera-Tomo 3D (OSEM), with corrections for depth-of-interaction (DOI), radionuclide decay, randoms, crystal dead time, detector normalization, and attenuation correction, and with a detector coincidence mode of 1:3, 4 iterations and 6 subsets, no filtering. CT images were reconstructed using RamLak filter. The PET and CT images were co-registered automatically. Images were reconstructed with a voxel size of 0.25×0.25×0.25 mm^3^ for CT, and 0.4×0.4×0.4 mm^3^ for PET. Data analyses were performed using InterView Fusion version 2.02.055.2010 (Mediso Ldt., Budapest, Hungary). For each scan a spherical volume of interest (VOI) with radius 1.5 mm were drawn manually over the muscles in the back of the neck. Standard uptake value (SUV) was calculated using the equation: SUV = C_PET_(T)/(ID/BW), where C_PET_(T) is the measured activity in tissue, ID is injected dose measured in kBq, and BW is mouse body weight in kg. SUV_mean_ is the SUV mean value of all voxels included in the VOI. SUV_mean_ in this nuchal muscle was used as a reference tissue in order to enable segmentation of putative tumour tissue having SUV ratios (SUVR) of >2 and >6 for ^18^F-FLT and ^18^F-FDG, respectively. VOIs of primary uterine tumours and of likely metastases were drawn semi-automatically in the PET images for estimation of metabolic tumour volumes (MTV) and their corresponding SUV_mean_. The parameter Total Lesion Glycolysis (TLG) in the tumour was calculated based on the ^18^F-FDG PET-CT scans using the following equation: TLG = FDG-SUV_mean_ x MTV [22]. For the ^18^F-FLT PET-CT scans a similar parameter was calculated based on the same equation and named FLT-SUV_mean_ x MTV.

#### MRI

MRI was performed in one mouse at week 11 after injection of Ishikawa^Luc^ cells (3 weeks before sacrifice). The MRI scan was performed on a 7T horizontal-bore preclinical scanner (Pharmascan 70/16, Bruker Corporation, Germany), using a 40 mm ID mouse body quadrature volume resonator in a single-coil (TX/RX) configuration. During scanning, the mouse was anesthetized using 3.0% sevoflurane. Respiration rate and body temperature were monitored and kept constant at 60±20 respiratory cycles/min and 37^+^/-2°C, respectively. For identification of tumour and estimation of tumour size, a *T*_2_-weighted rapid acquisition with relaxation enhancement (RARE) sequence (TE/TR = 36/4300 ms, 2 averages, matrix 256x256, field of view (FOV) 3.2 x 3.2 cm, slice thickness 1 mm) and pre- and a post-contrast *T*_1_-weighted RARE sequences (TE/TR = 9/1000 ms, 4 averages, matrix 256x256, FOV 3.2 x 3.2 cm, slice thickness 1 mm) were employed. The post-contrast images were collected after intravenous tail-vein injection of Gd-based contrast agent (Dotarem, Guerbet USA, volume 30uL, dose 0.1 mmol/kg of body weight). In addition, apparent diffusion coefficient (ADC) maps were generated from diffusion-weighted EPI images (DWI) (TE/TR = 24.6/3100 ms, 2 averages, matrix 128x128, FOV 3.2 x 3.2 cm, slice thickness 1 mm and 3 diffusion directions with b-values of 100, 200, 400, 600, 800, 1000 s/mm^2^).

### Histological examination and Immunohistochemical (IHC) analysis

Formalin-fixed tissue was processed for routine histological examination. 4 μm sections were stained with Hematoxylin–Eosin (HE) and examined by a pathologist (NCMV) for typing and grading of the tumours. To verify presence of human cells, IHC staining was performed on full section to detect expression of human ERα. Briefly, sections were dewaxed with xylene, rehydrated in graded ethanol before microwave antigen retrieval, and stained for 60 min in room temperature for human ERα expression using 1:400 HC-20 (Santa Cruz Biotechnology, Dallas, TX, USA) or 1:400 Clone SP1 (Thermo Scientific, Fremont, CA, USA). Anti-rabbit secondary antibody (Dako, Denmark) was applied for 30 minutes, followed by 8 minutes with Diaminobenzidine (DAB+, Dako, Denmark) before counterstaining with hematoxylin. To verify the species specific nature of the antibody, sections were compared to the non-species specific antibody from Santa Cruz (S1 Fig), validating that the human specific antibody only detected ERα in cells of human origin.

## Results

### Ishikawa^Luc^ cells form primary endometrial cancers in mice

**Fig 1:**
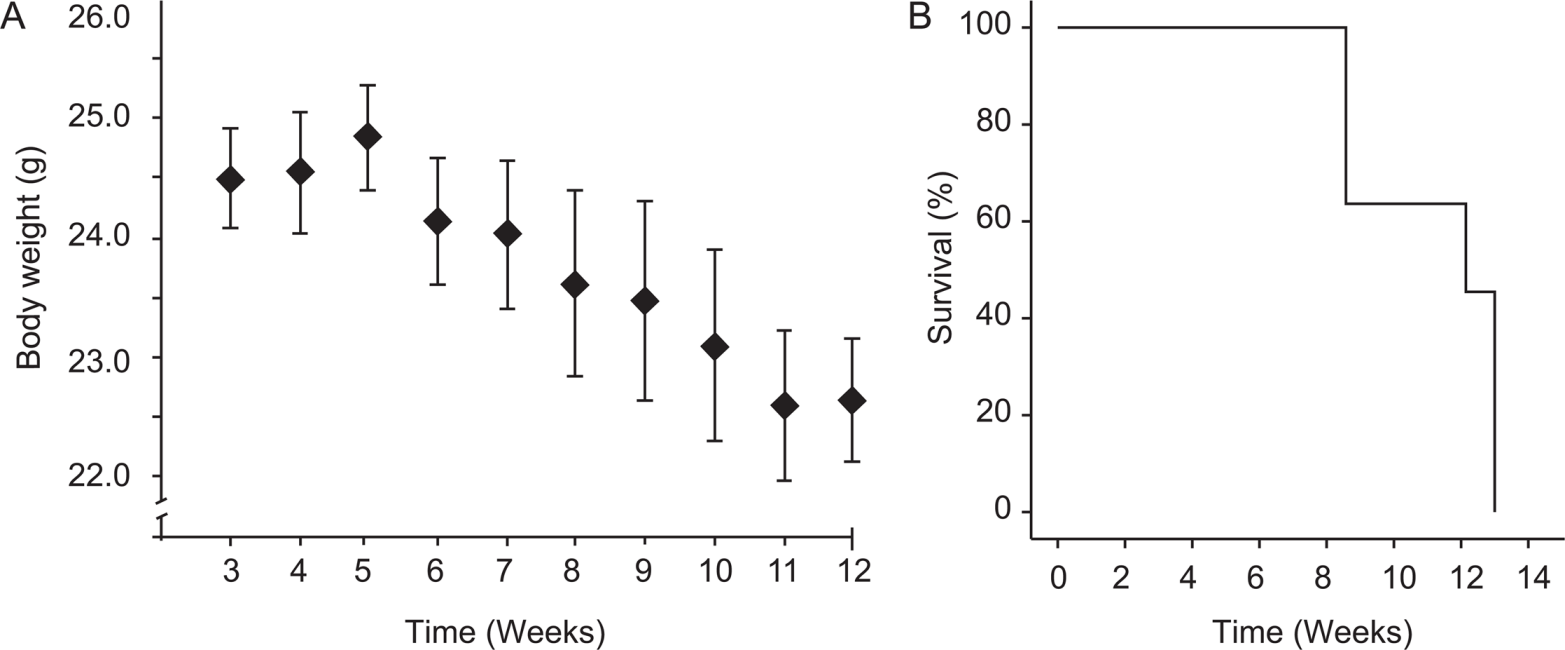
Orthotopic injection of Ishikawa^Luc^ cells results in weight loss and reduced survival. Mice injected with Ishikawa^Luc^ cells were monitored weekly for signs of disease development. Weight loss (A) was detected as an early sign of disease. Mice developing symptoms of severe disease were sacrificed and the overall survival is visualized in a Kaplan-Meier survival plot (B).

We developed an orthotopic mouse model that can be monitored by bioluminescence. Mice were monitored for up to 13 weeks and sacrificed when reaching humane endpoint. Body weight was monitored weekly (Fig 1A). A clear reduction in body weight was observed as the BLI signal increased. At 13 weeks post injection, all mice had reached a moribund disease condition (Fig 1B).

### Bioluminescence monitoring of tumour growth

**Fig 2:**
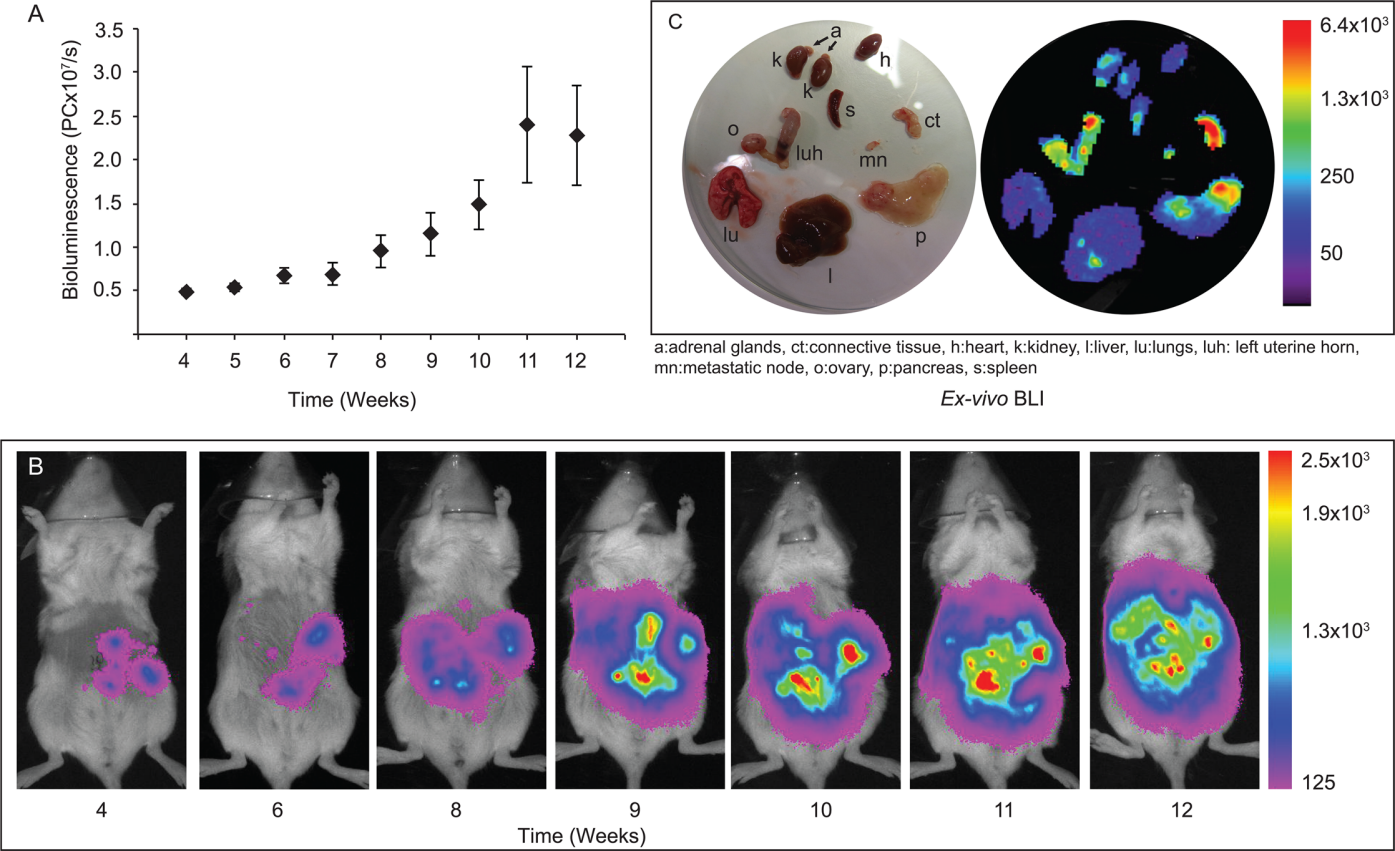
Tumour growth monitored by Bioluminescence Imaging (BLI). Tumour growth was monitored weekly by *in vivo* BLI and an increase in the net bioluminescence versus time was observed (A, B). Organs were also examined by BLI post-mortem to visualize metastatic spread (C). Strong BLI signals were detected at site of injection (left uterine horn; luh), right ovary (o), connective tissue surrounding the uterine horn (ct), pancreas (p) and metastatic node (mn). Spot signals were detected in the liver (l), spleen (s), kidneys (k), heart (h) and lung (lu). No signal was detected in adrenal gland (a).

A bioluminescence signal restricted to the uterine area was observed the first weeks, followed by more diffuse abdominal signal as the tumour burden was increasing and the mice developed metastatic disease. The total BLI signal increased dramatically the last weeks before the animals were sacrificed (Fig 2A and 2B).

Two mice showed no signs of metastatic spread but had large tumours limited to the uterus when sacrificed. The remaining nine mice had all developed advanced disease with metastatic spread and variable amounts of ascites. After macroscopic post-mortem examination, organs were imaged *ex vivo* to detect tumour cell dissemination (Fig 2C, Table 1). A strong BLI signal was observed in the uterus of all animals. Increased BLI signal was observed in the ovaries (n = 4), pancreas (n = 7), kidney (n = 2), spleen (n = 3), liver (n = 6) and lung (n = 4) as well as in connective tissue surrounding the uterus (n = 9). Nodules suspected to be metastatic lymph nodes were also BLI positive (n = 4). No BLI signal was detected in the adrenal glands.

**Table 1:** Tumour development and metastasis dissemination in Ishikawa^Luc^ model. *Total number of mice with organs affected by disease*, *defined by positive BLI signal and presence of cancer cells in histologic sections*.

Organ affected:	BLI (%)	Histology (%)
Uterus	11 (100)	11 (100)
Mice with metastases	9 (82)	9 (82)
Ascites	8 (73)	NA
Lymph nodes	4 (36)	4 (36)
Ovaries	4 (36)	4 (36)
Liver	7 (64)	4 (36)
Lungs	4 (36)	6 (55)
Connective tissue	9 (82)	9 (82)
Pancreas	7 (64)	7 (64)
Spleen	3 (27)	0
Kidneys	2 (18)	0
Adrenal glands	0 (0)	0 (0)

*NA*: *Not applicable*.

### Histological evaluation of the model

**Fig 3:**
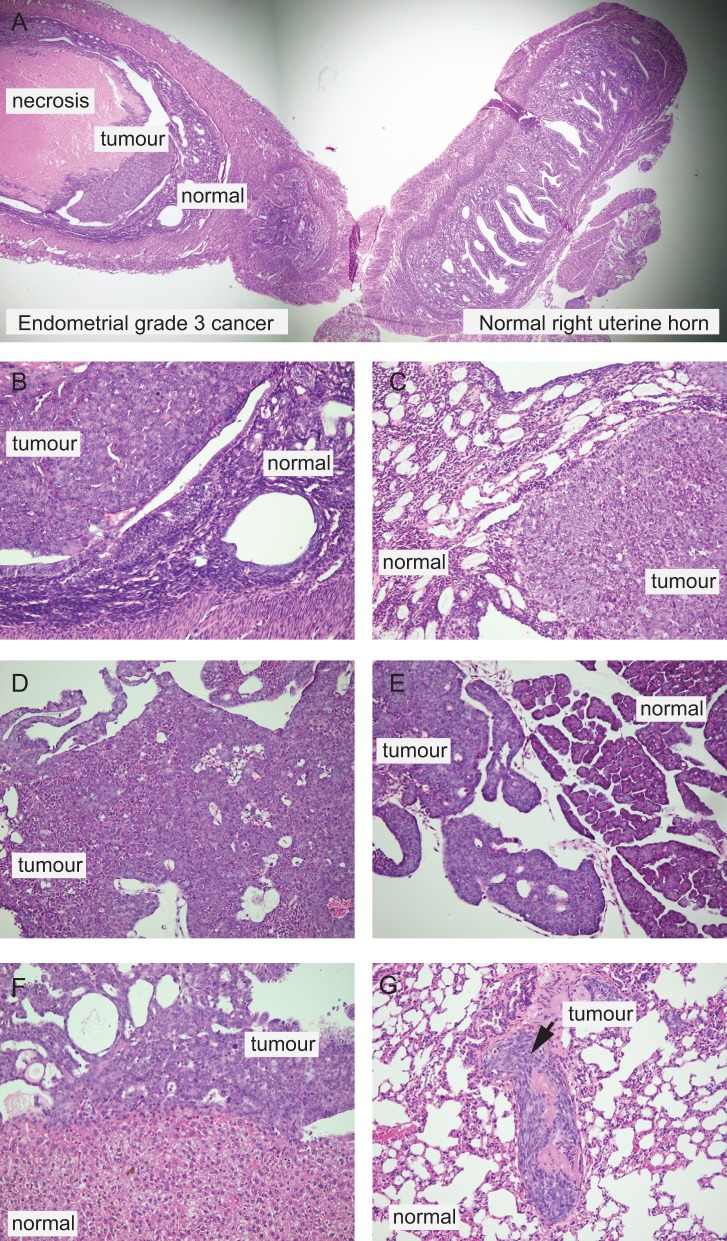
Histological evaluations of tumour characteristics and spread of disease. Organs were fixed in formaldehyde, sectioned and stained with HE to confirm presence of tumour tissue and for histological characterization of tumour. Sections from a representative mouse depict a large tumour mass in the left uterine horn (A) with necrotic tissue in the centre. Normal uterine morphology is seen in the right uterine horn with endometrial glands and normal stroma and myometrium. Detail of tumour in the left uterine horn (B) reveals solid growing tumour, resembling a grade 3 endometrioid endometrial cancer. Solid tumour masses were also detected in ovaries (C). Inguinal lymph node, macroscopically suspected to be metastatic, was confirmed to represent a metastasis (D), however, without visible surrounding lymphoid tissue. Solid tumour components are depicted in the pancreas (E) with tumour tissue infiltrating surrounding fat tissue. Metastasis is observed on the outer surface of the liver (F), and tumour tissue is also detected in blood vessels of the lung (G), the latter indicating hematogenous spread.

The histology of the primary tumour and presence of metastatic spread were determined on HE stained sections (Fig 3). Histological evaluation of the organs revealed normal histology with intact endometrial glands in the right uterine horn. A solid growing primary tumour was detected in the left uterine horn (at site of injection; Fig 3A) with characteristics of a grade 3 endometrioid endometrial cancer (Fig 3B), and areas of necrosis and myometrial invasion. Metastases to the ovaries (Fig 3C) were mostly solid with necrotic areas, but gland formation was also detectable in some cases. No remaining lymph node tissue was detected in metastatic nodes (Fig 3D). Larger separately growing tumour fragments were detected in the pancreas (Fig 3E). BLI positive kidneys and spleens had tumour cells in the outer surface of the organ, with no invasion of the parenchyma. This was also true for three BLI positive liver biopsies (Fig 3F). All mice with cancer cells detected in the outer lining of organs had ascites. Diffuse small tumour nodules were found in vessels in the lungs (Fig 3G), also in two lung biopsies that appeared negative in the BLI imaging (Table 1).

Cellular protein expression of human ERα was verified in human cells (S2 Fig). We found heterogeneous expression of ERα in the primary tumour, also showing myometrial infiltration (S2A Fig), consistent with reports that Ishikawa cells are ERα positive but with a tendency to lose expression of ERα with dedifferentiation. We also clearly identified presence of metastatic Ishikawa^Luc^ cells in pancreatic tissue (S2B Fig), in the capsule of the liver (S2C Fig) and in the vessels in pulmonary tissue (S2D Fig).

### PET-CT monitoring of tumour progression

**Fig 4:**
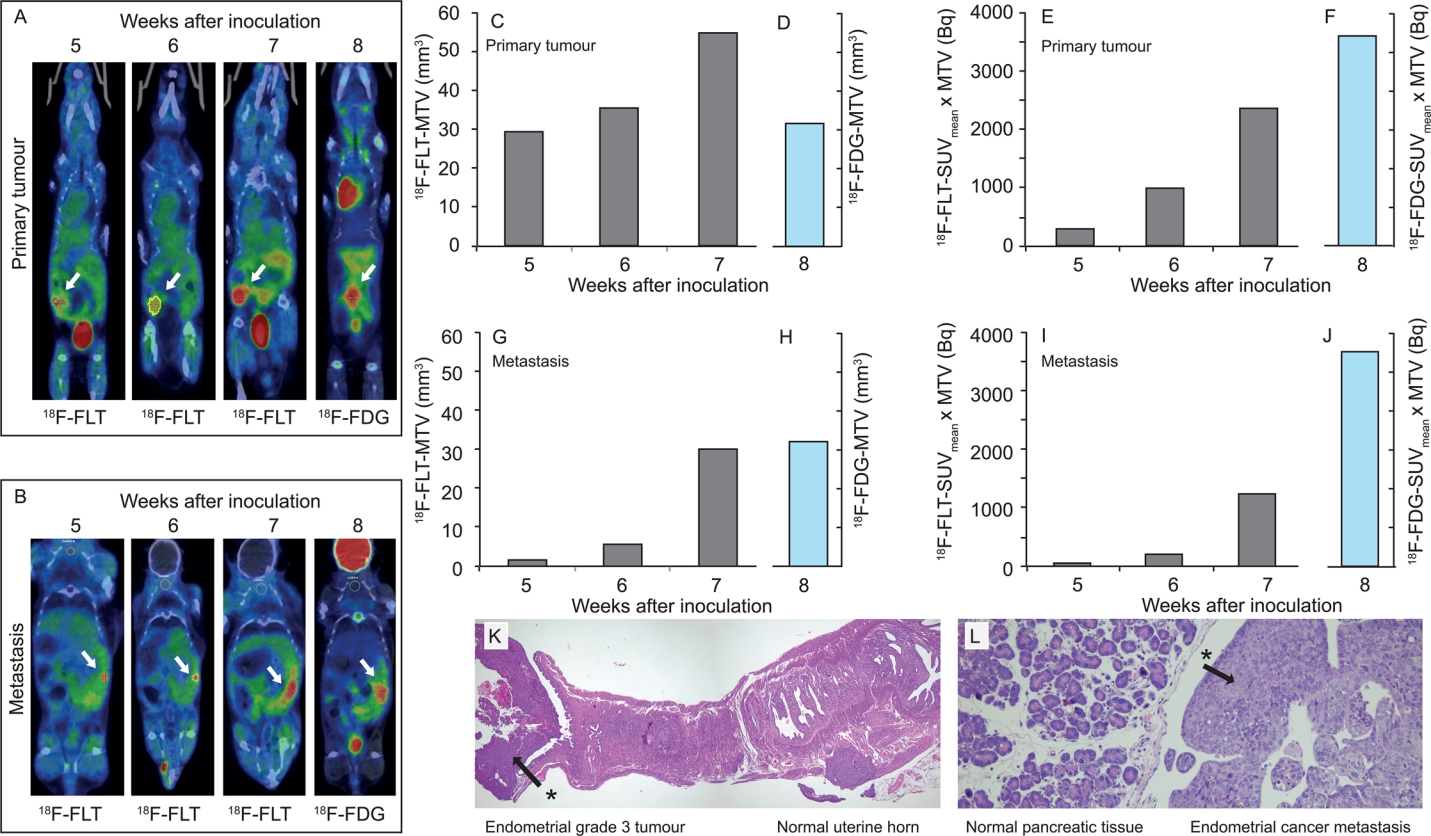
Tumour growth monitored by PET-CT. Tumour growth in the left uterine horn (A) and growth of abdominal metastasis (B) measured by ^18^F-FLT PET-CT at 5, 6 and 7 weeks after inoculation of cells; and by ^18^F-FDG PET-CT 8 weeks after inoculation (A and B) in the same mouse. Estimated metabolic tumour volume increased from 5 to 7 weeks after inoculation based on ^18^F-FLT PET-CT but was stable or slightly decreased from 7 to 8 weeks after inoculation based on ^18^F-FLT PET-CT (week 7) and ^18^F-FDG PET-CT (week 8) (C/D, G/H). The estimated ^18^F-FLT-SUV_mean_ x MTV steadily increased from 5 to 7 weeks after inoculation in both the primary tumour (E) and in the metastasis (I). Panel F and J show Total Lesion Glycolysis (^18^F-FDG-SUV_mean_ x MTV) for primary tumor and metastasis, respectively. Histologic examination of the uterus (K) and the pancreas (L) validated presence of malignant tissue (asterisks) as detected with PET-CT.

**Fig 5:**
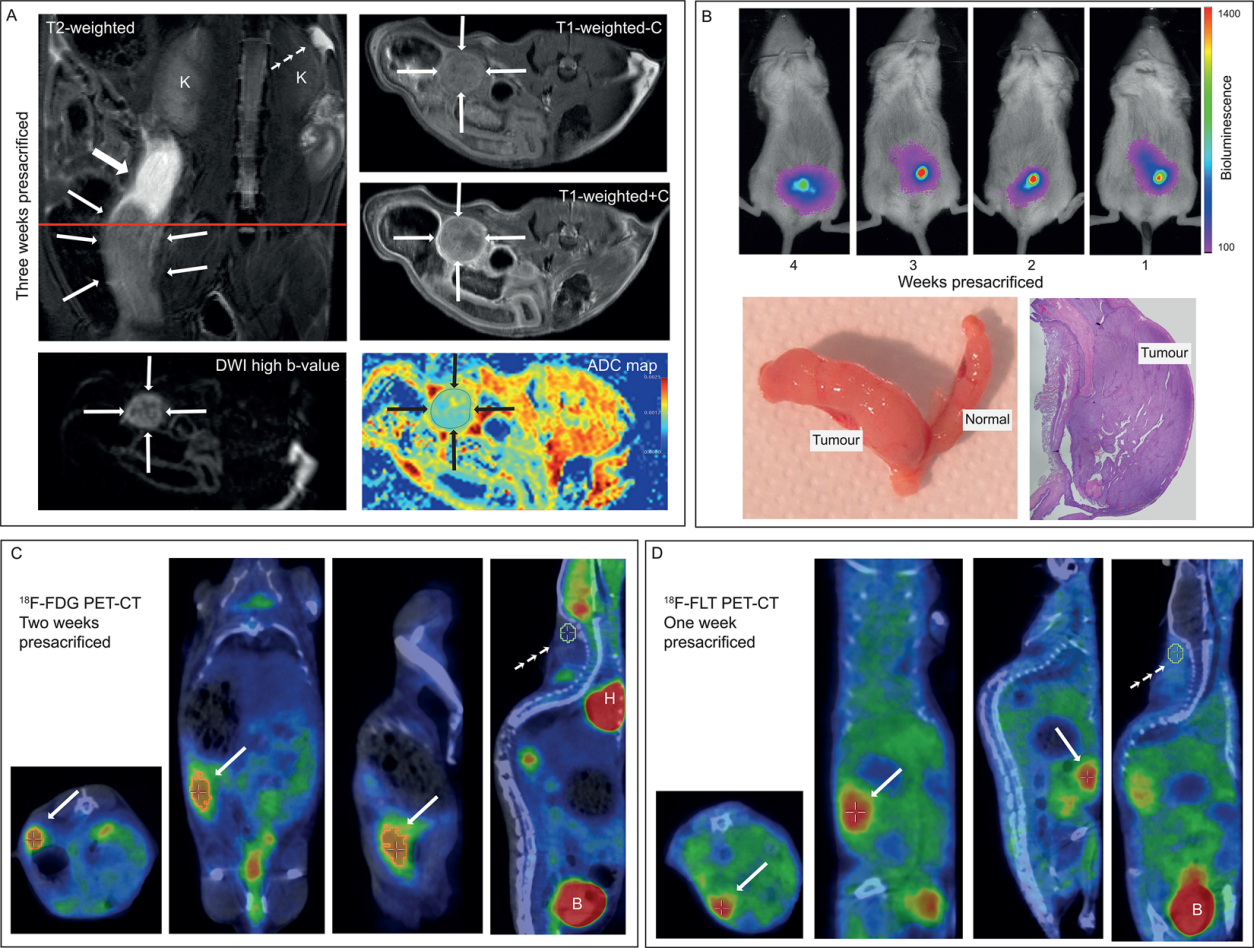
Multimodal imaging of the same mouse by MRI, ^18^F-FDG PET, ^18^F-FLT PET and BLI. MRI three weeks presacrificed (A) depicting large uterine tumour tissue in the left uterine horn (thin arrows) with intrauterine fluid cranial of the tumour (filled large arrow) and small amounts of free intraperitoneal fluid cranial to the right kidney (K) (small arrows). The tumour tissue is moderately enhancing on T1-weighted series after contrast and the tumour exhibits restricted diffusion with hyperintensity on high b-value DWI with corresponding low apparent diffusion coefficient (ADC) value (1.11 x 10^−3^ mm^2^/s) on the ADC map (A). BLI 4 to 1 weeks presacrificed (B) shows increasing BLI signal corresponding to the tumour of the left uterine horn; the corresponding tumour tissue was evident macroscopically and confirmed microscopically at necropsy (B). ^18^F-FDG PET-CT two weeks presacrificed (C) depicts a large ^18^F-FDG-avid tumour in the left uterine horn (arrows) with estimated metabolic tumour volume of 33 ml. ^18^F-FLT PET-CT one week presacrificed (D) depicts large ^18^F-FLT-avid tumour in the left uterine horn (arrows) with estimated metabolic tumour volume of 44 ml. ^18^F-FDG/^18^F-FLT-avidity in a VOI in the nuchal muscular tissue (C and D; small arrows) was used as reference tissue to define a threshold for likely tumour tissue (activity of x2 and of x6 for ^18^F-FLT and ^18^F-FDG, respectively) to be included in the estimated metabolic tumour volume. B: bladder; H: heart.

Evident ^18^F-FDG/^18^F-FLT-avid tumour tissue in the uterus (Fig 4A and 4B and Fig 5C and 5D) was observed in 6 out of 7 mice, which were scanned ≤ 35 days prior to sacrifice. The remaining mice (n = 4) showed no visible tumour at their last PET-CT scans, 49 days prior to sacrifice. However, these mice did develop tumour growth and metastases at a later point with increased BLI tumour signal after their last PET-CT scans.

In the mice having weekly PET-CT scans the last weeks before sacrifice, increasing MTV and SUV_mean_ x MTV was observed in both primary tumours (Fig 4A and 4C–4F) and metastases (Fig 4B and 4G–4J). The highly ^18^F-FDG/^18^F-FLT- avid tissue (Fig 4A and 4B), assumed to represent tumours, was histologically confirmed as primary malignant uterine tumours (Fig 4K) and metastases (Fig 4L), respectively, at necropsy. Although the absolute values for calculated MTV and SUV_mean_ x MTV of the tumours using different tracers on PET-CT were not directly comparable, both tracers seemed equally feasible to depict tumour tissue and monitoring tumour growth and metastatic spread at PET-CT (Fig 4A and 4B and Fig 5C and 5D).

### MRI of tumour model

MRI conspicuously depicted the boundaries of the uterine tumour (Fig 5A). The tumour was hyperintense on T2-weigthed images and moderately contrast-enhancing on T1-weighted series after i.v. contrast. Restricted diffusion within the tumour tissue was striking with hyperintensity on high b-value images and corresponding low ADC value on the ADC map (Fig 5A).

### The PET-CT and MRI findings of the Ishikawa^Luc^ cell model are similar to patterns detected for human endometrial cancers

The uterine tumours in the mice were highly ^18^F-FDG-avid (Figs 4A and 4B and 5C), thus resembling the metabolic behaviour of the human endometrial cancers, which also typically exhibit marked ^18^F-FDG-avidity (S3A, S3E Fig). Both in patients and in the preclinical model, the tumour is hyperintense on T2-weighted images (Fig 5A and S3C, S3F Fig) and moderately contrast-enhancing on T1-weighted images after intravenous contrast (Fig 5A and S3D Fig). Similarly, the restricted diffusion observed within the tumour in the mouse model (with measured tumour ADC value of 1.11 x 10^−3^ mm^2^/s; Fig 5A) is quite similar to that in endometrial cancer tissue in patients (S3G and S3H Fig; tumour ADC value in this patient was 0.83 x 10^−3^ mm^2^/s).

### PET-CT represents a powerful tool for detection of tumour tissue in PDX model of endometrial cancer

**Fig 6:**
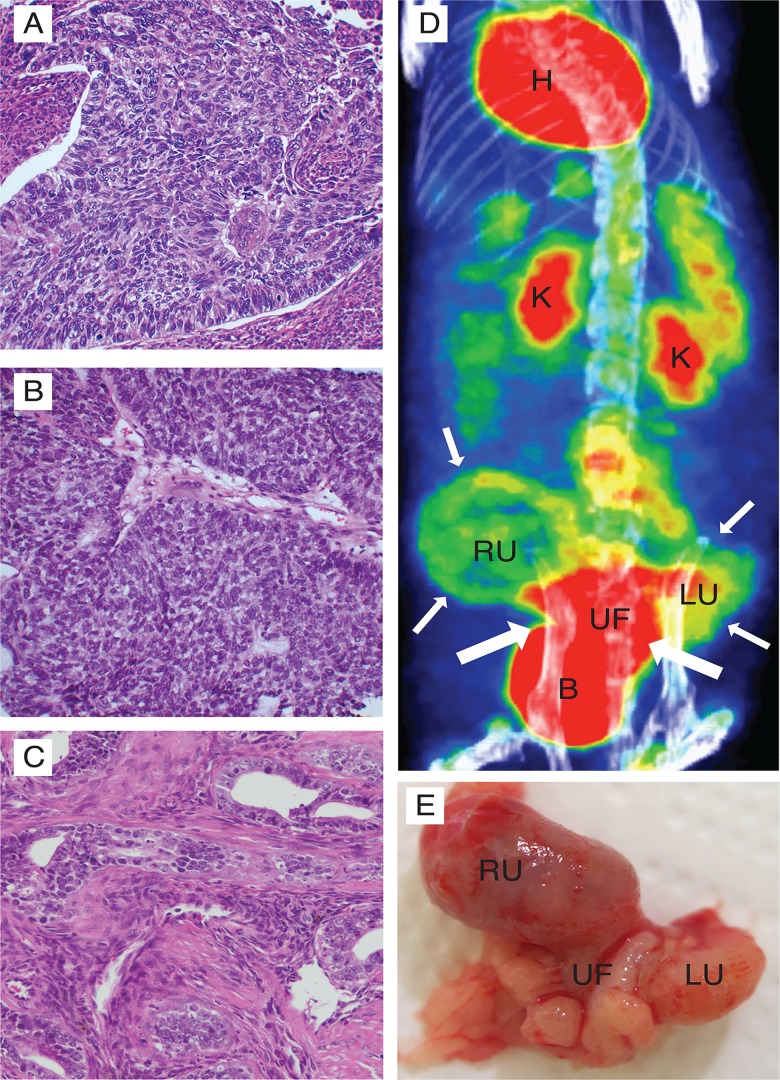
^18^F-FDG PET detects tumour in an orthotopic endometrial cancer PDX model. Mice were implanted in the uterus with cancer cells from a patient biopsy and rederived for two generations to develop a PDX model. Histological examination revealed that both the F1 (B) and the F2 (C) mice developed tumours closely resembling the parental tumour, defined as an endometrioid grade 3 endometrial cancer (A). ^18^F-FDG PET was successfully used to detect tumour growth in both generation F1 (not shown) and generation F2 (D). Highly ^18^F-FDG-avid tumour tissue (large arrows) in the uterine fundus (UF) and both the left and the right uterine horn (LU, RU respectively) depicted at PET-CT (D). Macroscopic examination revealed a large tumour infiltrating both the uterine horns as well as the bladder (E) and corresponded well with the tissue detected as tumour by ^18^F-FDG PET. B, bladder; H, heart; K, kidney; LU, left uterine horn; RU, right uterine horn; UF, uterine fundus.

Although orthotopic models generated from established cell lines develop tumours in the uterus, a PDX model is likely to better mimic the clinical setting relevant for disease spread and response to therapy. This PDX model was developed from a patient primary tumour (Fig 6A) with grade 3, endometrioid endometrial type. Histologic examination of the primary tumours of both the F1 (Fig 6B) and the F2 (Fig 6C) generation reveal high resemblance to the donors, and both were classified as grade 3 endometrioid, endometrial cancers by the pathologist. PET-CT was successfully used to detect tumour growth using ^18^F-FLT in generation F1 (not shown) and ^18^F-FDG in generation F2 (Fig 6D). Highly ^18^F-FDG-avid tumour tissue in the uterine fundus and both the left and the right uterine horn was macroscopically verified to represent tumour tissue (Fig 6E).

## Discussion

One major challenge in cancer research today is to what extent the cancer models mimic the disease in a human setting. The use of mouse models has become increasingly popular, including both sub-cutaneous and orthotopic models, in combination with cell line based and PDX models [23]. The ability to monitor disease development and response to therapy, is of great importance when choosing the model system, and several non-invasive imaging models are presently available, although not extensively explored for uterine cancer. Still, many of these are best suited for cell line based models since they ultimately rely on genetic engineering of the cells. The availability of PET tracers and small scale imaging equipment for PET-CT and MRI has improved our ability to study cancer development and metastatic spread without such use of reporter genes [24]. This study, describing the typical imaging findings based on *in vivo* BLI in parallel with PET-CT and MRI findings for the first time in an orthotopic endometrial cancer model, demonstrates an excellent feasibility of this multimodal imaging platform to monitor tumour progression and metastatic spread.

We utilized the orthotopic Ishikawa^LUC^ model of endometrial cancer to trace tumour growth using BLI and verified the methods ability to detect tumour growth and spread by macroscopic and microscopic necropsy examination of affected organs. As reported previously by others [7, 21, 25], BLI proved to be an effective imaging method for detection of tumour growth and metastases, especially when combined with *post mortem ex vivo* BLI imaging of organs. For growth of tumour cells in the peripheral margins of an organ, the *in vivo* BLI method was not able to accurately detect the exact organ specific location of the signal and *ex vivo* BLI or microscopic examination was necessary. In spite of these limitations, BLI is a powerful imaging tool to monitor cancer cell line growth, however with limited value for monitoring tumour growth in orthotopic PDX models. We therefor explored the same mice in parallel by PET-CT and MRI to explore the potential for these methods to detect tumour development.

PET-CT was performed using two different tracers enabling imaging of different metabolic properties of the tumour tissue. ^18^F-FLT is a nucleoside analogue that is taken up in proliferating cells in the S-phase. ^18^F-FLT enters cells via passive diffusion and active nucleoside transporters. It undergoes phosphorylation by the enzyme thymidine kinase 1 (TK1) and is trapped intracellularly [26]. The uptake of ^18^F-FLT is thus related to the metabolism of the nucleosides and is considered a marker for cell proliferation [16]. ^18^F-FDG is a glucose analogue that tends to accumulate in tissue with upregulated glucose transporter expression and/or increased metabolic activity [16]. Most cancers show an increased aerobic glycolysis leading to an increased uptake of ^18^F-FDG. Whereas ^18^F-FDG-avidity is nonspecific for tumour tissue, and a characteristic also of inflammatory or infectious disease, ^18^F-FLT-avidity is believed to almost uniformly indicate presence of viable tumour cells [16]. Interestingly, both tracers seemed equally feasible of depicting and monitoring tumour growth and metastatic dissemination in this mouse model.

The uptake of both ^18^F-FDG and ^18^F-FLT in a tumour is considered to reflect tumour viable cell densities, and the product SUV_mean_ x MTV, referred to as Total Lesion Glycolysis when using ^18^F-FDG as tracer, is thus a measure of the total number of viable tumour cells in the tumour [22]. Interestingly, we observed a gradual increase in SUV_mean_ x MTV prior to sacrifice both in the primary tumour and in the metastasis (Fig 4E/4F and 4I/4J, respectively), whereas estimated MTV in a mouse decreased in the primary tumour and was stable in the metastasis at the last study when ^18^F-FDG replaced ^18^F-FLT (Fig 4C/4D and 4G/4H, respectively). A plausible explanation of this finding regarding estimated MTV when using different tracers may be differences in tracer characteristics for the same tumour, as well as the applied thresholds for the two tracers to estimate MTV. Further studies are needed to establish optimal PET imaging parameters and PET tracers for reliable estimation of tumour burden and progression as well as response to therapy in this mouse model.

The ability of PET-CT to validly detect and to monitor tumour growth quantitatively is affected by various known factors such as tumour size, surrounding background activity, spatial resolution of the PET-CT scanner and reconstruction algorithms of the scanner [27]. The recorded spatial resolution of 800 μm and 30 μm for the respective PET- and CT detector systems of the PET-CT scanner in this study, would ideally allow detection of very small lesions down to 0.8 mm. However, we found that early detection of the very small uterine tumours and metastases were difficult due to radiotracer uptake in neighbouring organs i.e. the bladder and kidneys and often also the intestines. However, when a gradual increase of radiotracer activity was depicted in the uterus and at the same extrauterine sites on consecutive images (suggesting metastases), the evidence of tumour growth seemed obvious, and the tissue of increased tracer uptake was confirmed to represent tumours at the corresponding sites at necropsy.

A limitation of this study is its ineligibility to compare the estimated MTV at PET to tumour volumes based on necropsy, which would be highly interesting. Such a comparison would allow an analysis of the optimal threshold for discriminating the tumour from the surrounding tissue and for estimating the corresponding correct tumour volumes. However, in order to perform such a comparison meaningfully, the PET-CT should be performed immediately prior to sacrifice. Since this was not done in the present study, we plan to do so in a follow-up study in order to further refine and validate the MTV measurements based on PET-CT.

The imaging findings presented in this study during tumour growth and metastatic spread apply to untreated mice. Future studies will thus be needed to explore the feasibility of the same multimodal imaging platform to evaluate treatment response during therapy. However, the ability to visualize and quantify the tumor burden before treatment is a prerequisite in order to succeed in the evaluation of treatment response. Thus, we propose that the presented findings in untreated mice make the same imaging methods very promising for evaluation of treatment response, although this remains to be demonstrated in future studies. Furthermore, including preclinical ultrasound scanning during tumor growth and therapy may represent an intriguing addition to the multimodal imaging platform already explored in this study. Ultrasound may be especially translatable to the clinic, since patients with symptoms of endometrial cancer are often subjected to vaginal ultrasound as primary imaging examination.

Interestingly, the functional tumour characteristics based on PET-CT and MRI are very similar to that observed in human endometrial cancer, supporting a promising translational relevance of this imaging integrated tumour model for assessing tumour growth. The findings in this model derived from human endometrial cancer cell lines, as well as the PDX model, open the avenue for further exploring the value of PET-CT and MRI in monitoring tumour growth and metastatic spread in PDX models of endometrial cancer. This may prove highly clinically relevant since PDX models are likely to have an increased translational relevance for drug testing with relevance for a human tumour setting.

## Conclusion

We have demonstrated the feasibility of a multimodal imaging platform using BLI, ^18^F-FDG PET, ^18^F-FLT PET, and MRI to detect and monitor tumour progression in an orthotopic endometrial cancer model derived from human cell lines. The latter three imaging methods are well suited also in patients derived tumour models in which BLI is ineligible. PET-CT is also demonstrated to detect tumour tissue in a PDX model. PET-CT and MRI may thus represent powerful tools for monitoring tumour progression and functional changes for preclinical testing of systemic therapy in orthotopic models more reliably mimicking human endometrial cancer.

## Supporting Information

S1 Fig Identification of human specific anti-Estrogen Recetor α antibody. Immunohistochemical staining was performed according to a standard protocol. Paraffin sections from mouse uterus implanted with Ishikawa human endometrial cancer cells were stained with 1:50 anti-ERα **(**A; sc-543 Santa Cruz Biotechnologies) or 1:400 anti-ERα (B; SP1, Thermo) for detection of endometrial cells expressing ERα. Both antibodies are raised in rabbit and selected to avoid crossreaction with mouse immunoglobulins. The Santa Cruz antibody (A) was found to detect both mouse and human ERα, while the Thermo antibody SP1(B) was human specific. The SP1 antibody was selected to specifically detect localization and spread of implanted cells.
(PDF)

S2 Fig Detection of metastatic spread by IHC staining for human ERα. To verify presence of the ERα positive human Ishikawa^Luc^ cell line spread to distant organs, sections were stained for expression of human Estrogen Receptor α. Myometrial tumour infiltration was detected in the uterus (A) and metastases were detected in the pancreas (B), liver (C) and in the lungs (D); for all tumour sites positive staining for human ERα confirming spread of the human tumour cells.
(PDF)

S3 Fig Example of human endometrial carcinoma assessed by preoperative imaging and estrogen receptor staining in histological section. ^18^F-FDG PET-CT (A, E), CT (B), T2-weighed (C, F) and contrast enhanced T1-weighed (D) MRI, diffusion weighted imaging (*b* = 1000 s/mm^2^) (G) with corresponding apparent diffusion coefficient (ADC) map (H) and positive immunohistochemical staining for estrogen receptor of the uterine tumour tissue (I) from an 80-year old female with FIGO stage 2, endometrioid endometrial cancer. ^18^F-FDG PET-CT shows a highly ^18^F-FDG-avid uterine tumour (A, E; arrows) with an estimated metabolic tumour volume of 22 ml. The tumour is also conspicuously depicted at CT (B) and MRI (C-D, F-H; arrows) exhibiting restricted diffusion on the ADC map (H) with tumour ADC value of 0.83 x 10^−3^ mm^2^/s.
(PDF)
