# Supplementary figures and images for: Multimodal Imaging of Orthotopic Mouse Model of Endometrial Carcinoma
Source: PLoS One. 2015 Aug 7;10(8):e0135220. doi: 10.1371/journal.pone.0135220 (PMC4529312; doi:10.1371/journal.pone.0135220)

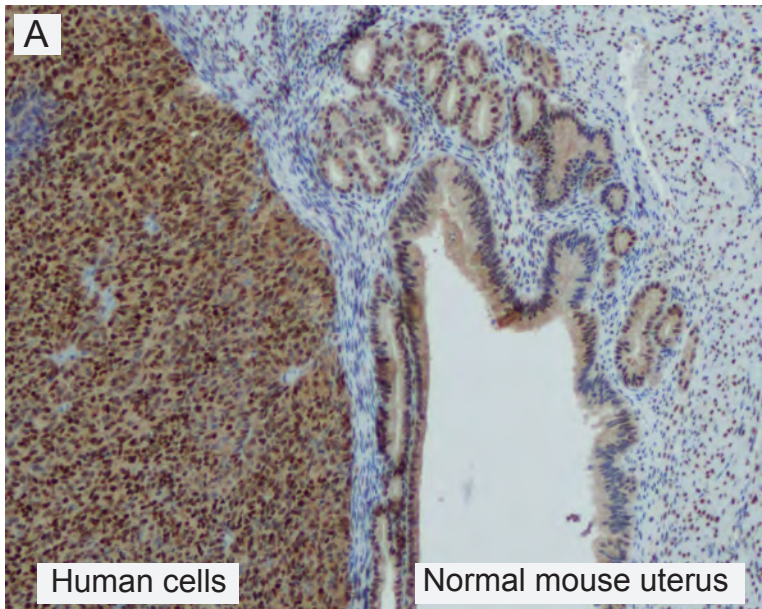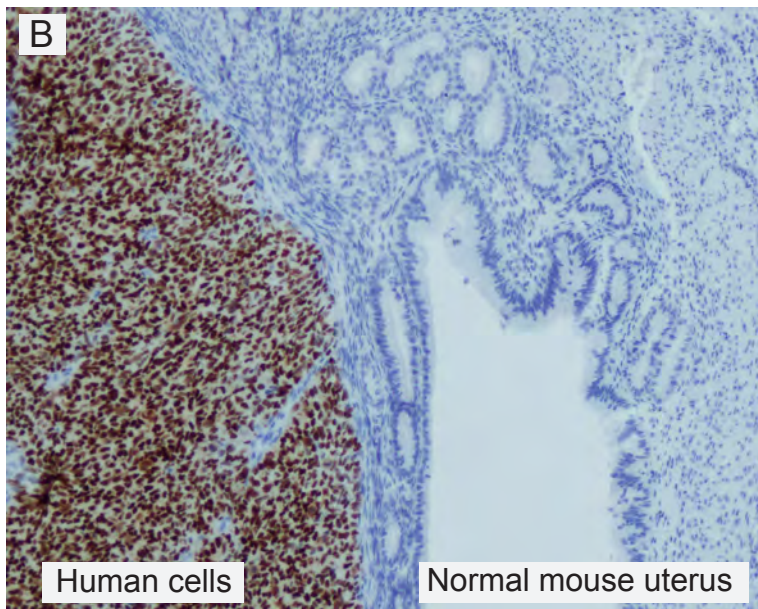

Supplement: S1 Fig — Immunohistochemical staining was performed according to a standard protocol. Paraffin sections from mouse uterus implanted with Ishikawa human endometrial cancer cells were stained with 1:50 anti-ERα (A; sc-543 Santa Cruz Biotechnologies) or 1:400 anti-ERα (B; SP1, Thermo) for detection of endometrial cells expressing ERα. Both antibodies are raised in rabbit and selected to avoid crossreaction with mouse immunoglobulins. The Santa Cruz antibody (A) was found to detect both mouse and human ERα, while the Thermo antibody SP1(B) was human specific. The SP1 antibody was selected to specifically detect localization and spread of implanted cells. (PDF) [file pone.0135220.s001.pdf]

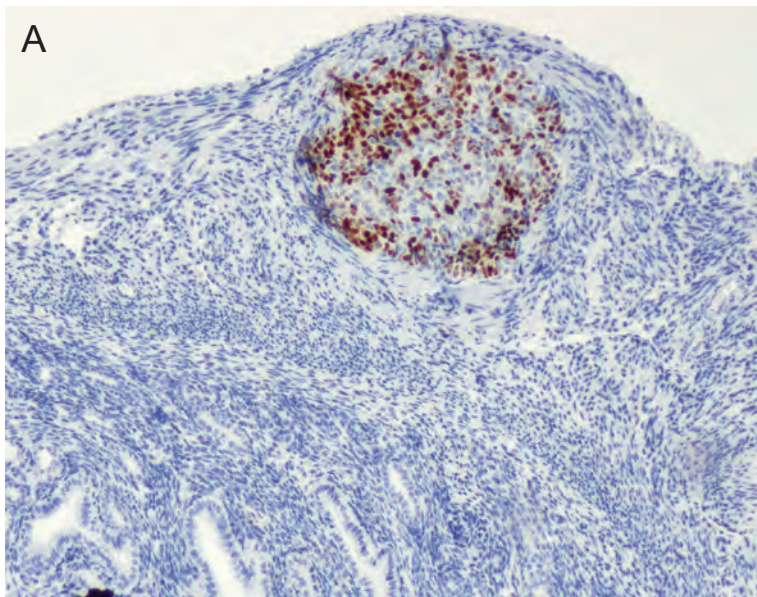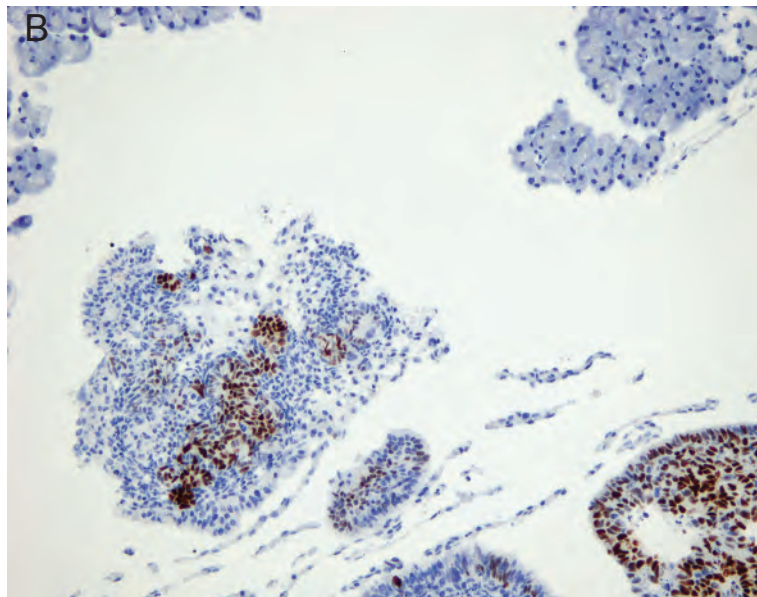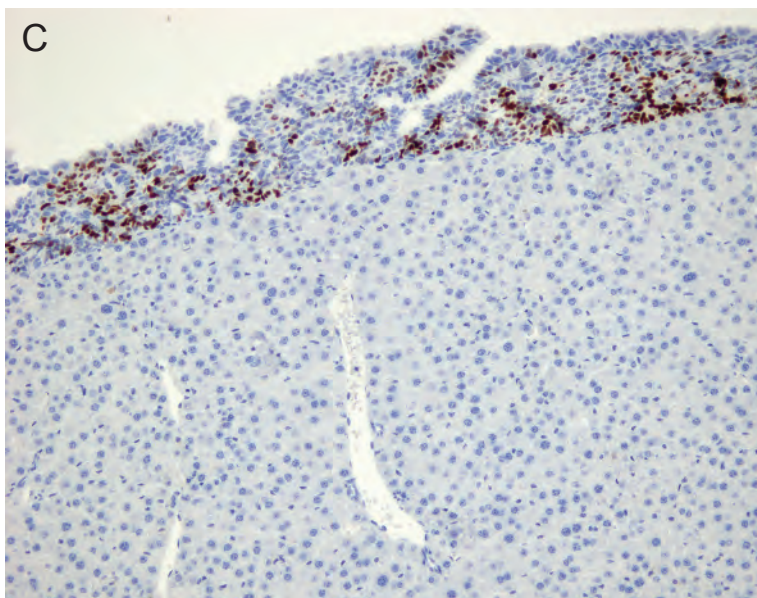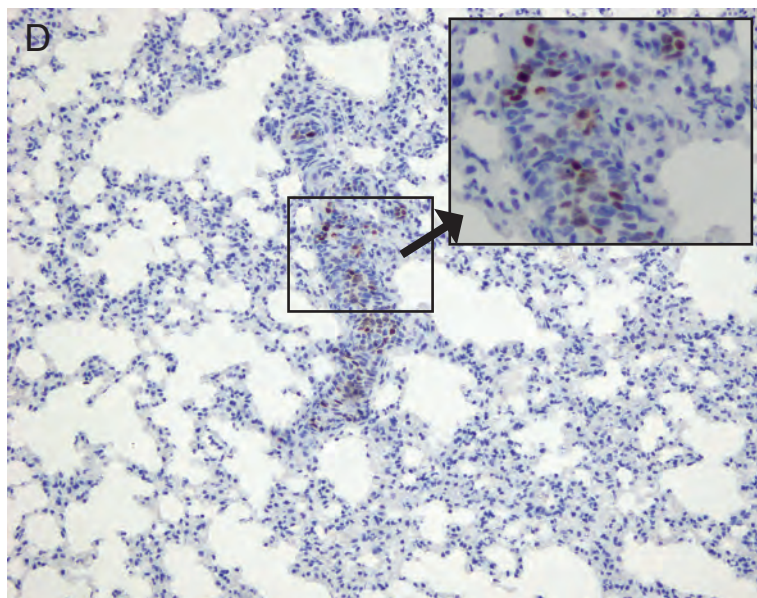

Supplement: S2 Fig — To verify presence of the ERα positive human IshikawaLuc cell line spread to distant organs, sections were stained for expression of human Estrogen Receptor α. Myometrial tumour infiltration was detected in the uterus (A) and metastases were detected in the pancreas (B), liver (C) and in the lungs (D); for all tumour sites positive staining for human ERα confirming spread of the human tumour cells. (PDF) [file pone.0135220.s002.pdf]

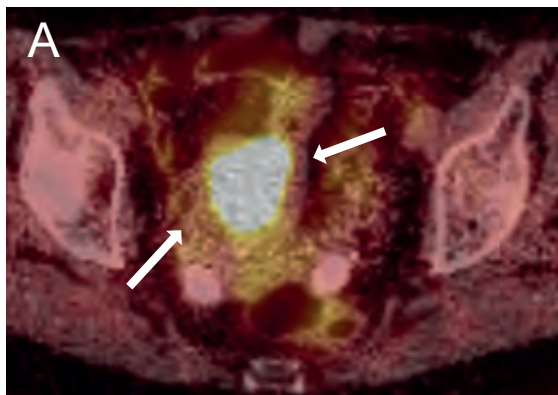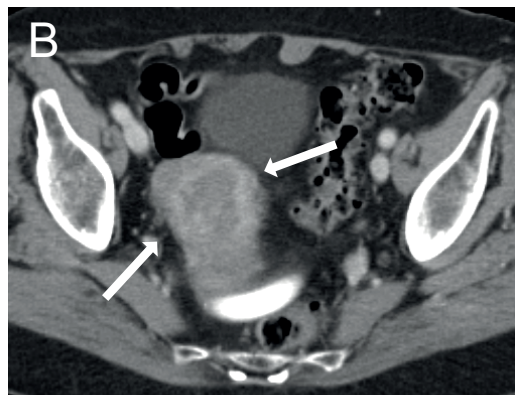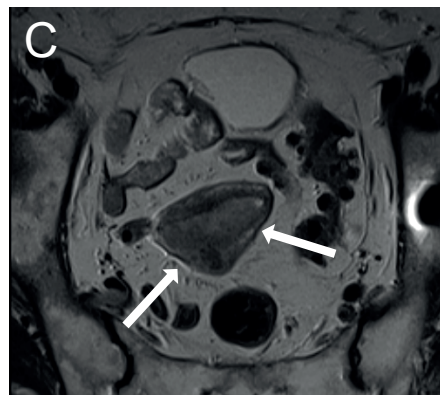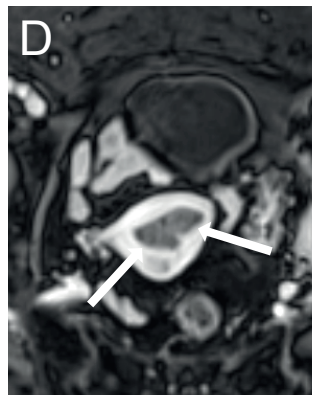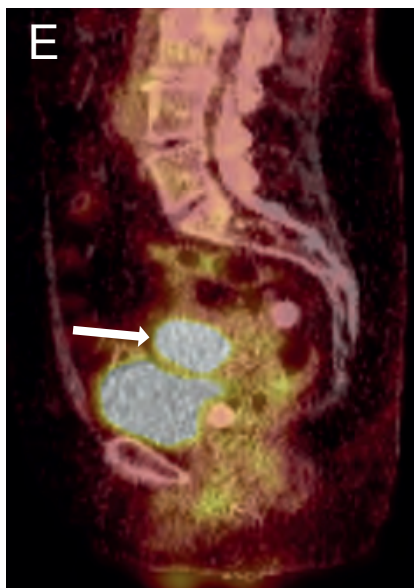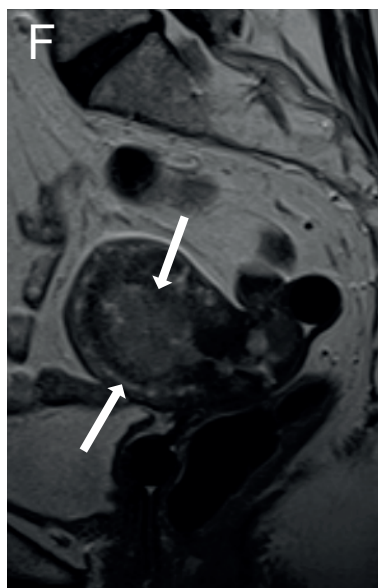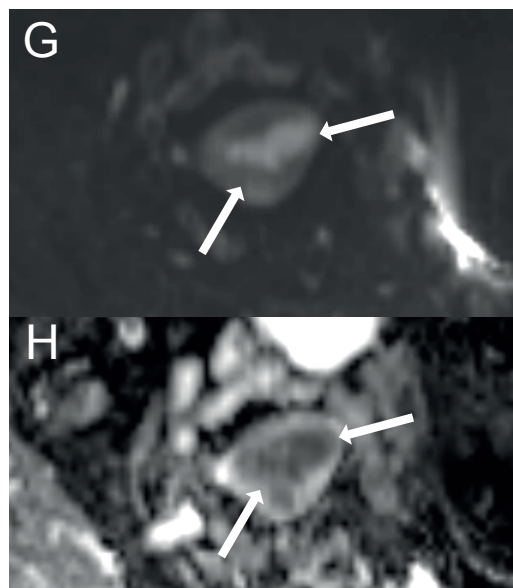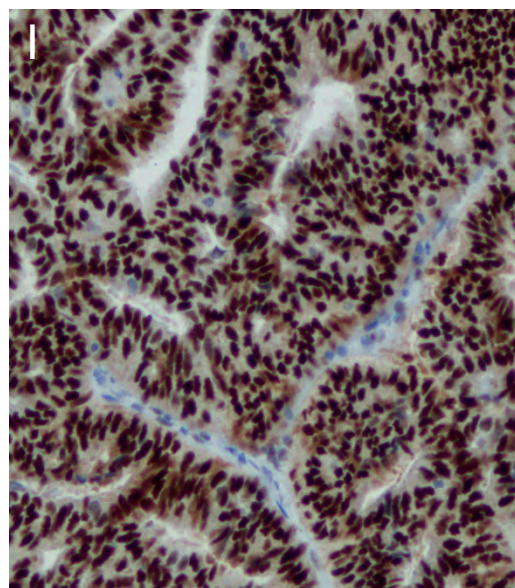

Supplement: S3 Fig — 18F-FDG PET-CT (A, E), CT (B), T2-weighed (C, F) and contrast enhanced T1-weighed (D) MRI, diffusion weighted imaging (b = 1000 s/mm2) (G) with corresponding apparent diffusion coefficient (ADC) map (H) and positive immunohistochemical staining for estrogen receptor of the uterine tumour tissue (I) from an 80-year old female with FIGO stage 2, endometrioid endometrial cancer. 18F-FDG PET-CT shows a highly 18F-FDG-avid uterine tumour (A, E; arrows) with an estimated metabolic tumour volume of 22 ml. The tumour is also conspicuously depicted at CT (B) and MRI (C-D, F-H; arrows) exhibiting restricted diffusion on the ADC map (H) with tumour ADC value of 0.83 x 10^−3^ mm2/s. (PDF) [file pone.0135220.s003.pdf]
